# Bushen Huoxue decoction alleviates bisphenol a-induced infertility through the PMK-1 mitogen-activated protein kinases signaling pathway and downstream mitochondrial unfolded protein response in *Caenorhabditis elegans*

**DOI:** 10.3389/fphar.2025.1713681

**Published:** 2026-01-15

**Authors:** Linlin Chen, Kanglu Wu, Chenyi Shou, Lijun Zhang, Mengya Tu, Borui Wang, Yun Cai, Zihui Zheng, Jia Sun, Qinli Ruan, Jun Guo

**Affiliations:** 1 School of Medicine, Nanjing University of Chinese Medicine, Nanjing, China; 2 School of Acupuncture-Moxibustion & Tuina, School of Health Preservation & Rehabilitation, Nanjing University of Chinese Medicine, Nanjing, China

**Keywords:** Bushen Huoxue decoction, fertility, metabolites combination, mitochondrial unfolded protein response, proteostasis, systemic stress resistance

## Abstract

**Introduction:**

Bushen Huoxue (BSHX) decoction is a traditional Chinese medicine formula that has been utilized clinically to treat Diminished Ovarian Reserve. However, the underlying mechanisms by which BSHX decoction increases fertility from the perspective of systemic stress protective responses remain poorly understood. This study aims to investigate how BSHX decoction improves female fertility by improving systemic stress resistance in a fertility-defective *Caenorhabditis elegans* model.

**Methods:**

Bisphenol A (BPA) was utilized to create a fertility-defective *C. elegans* model. Brood size was used to evaluate fertility. Survival under heat stress was used to evaluate stress resistance. Loss-of-function mutants and fluorescent protein transgenic strains were used to evaluate gene function. Polymerase chain reaction and RNA interference were used to detect gene expression levels or protein function.

**Results and Discussion:**

BSHX decoction significantly increased the fertility of BPA-exposed nematodes by inhibiting the accumulation of RHO-1 proteins in proximal oocytes. BSHX enhanced the heat stress resistance of germ cells, which is mediated by the PMK-1 and JNK mitogen-activated protein kinases (MAPK) pathways in germ cells. BSHX upregulated the transcriptional level and fluorescent protein level of the innate peptide T24B8.5 *via* the PMK-1 MAPK pathway. PMK-1 MAPK, induced by BSHX, further activated the mitochondrial unfolded protein response (UPR^mt^) in the gonad and intestine. The UPR^mt^ -regulated gene *hsp-6* was required to maintain mitochondrial function by reducing mitochondrial ROS levels and elevating the mitochondrial membrane potential, ultimately increasing the female fertility. In addition, a combination of metabolites (salvianolic acid B, quercetin, and asperosaponin VI), derived from the BSHX decoction, significantly enhanced the fertility of BPA-exposed nematodes through a mechanism highly similar to that of the BSHX decoction. Therefore, BSHX decoction increases fertility through the PMK-1 MAPK pathway and *hsp-6*-mediated UPR^mt^ in BPA-exposed *C. elegans.*

## 1 Introduction

The decline in female fertility is a significant social issue at present. Diminished ovarian reserve (DOR) affects approximately 10% of women experiencing infertility (Scott and Hofmann, 1995). It is characterized by a decline in both the quantity and quality of oocytes. A significant factor contributing to the decline in oocyte quality in DOR is disrupted protein homeostasis, which leads to the accumulation of misfolded or damaged proteins in proximal oocytes (Hart, 2016; Hipp et al., 2019).

Bushen Huoxue (BSHX) decoction, a traditional Chinese medicine formula recorded in *Essence of Famous Formulas from Chinese Gynecology Schools* (Hu and Luo, 2016), has been employed clinically to treat DOR and improve ovarian function (Xu et al., 2007). Both clinical and animal studies show that BSHX can regulate levels of key reproductive hormones (estradiol, follicle-stimulating hormone, and luteinizing hormone), and enhance follicle development (Xu et al., 2007; Xu et al., 2009).

The decoction includes the seed of *Cuscuta chinensis* Lam., the radix of *Rehmannia glutinosa* (Gaertn.) Libosch. ex DC., the radix of *Paeonia lactiflora* Pall., the radix of *Paeonia veitchii* Lynch, the fruit of *Cornus officinalis* Siebold & Zucc., the radix and rhizome of *Salvia miltiorrhiza* Bunge, the radix of *Dipsacus asper* Wall. ex DC. Among these Chinese herbs, *C. chinensis* induces the release of both interleukin-8 and MIP-1 beta (Shin et al., 2011). RNA-sequencing analysis of aged nematodes treated with *C. chinensis* indicates upregulation of genes involved in immune defense and protein homeostasis (Sayed et al., 2025). *Rehmannia glutinosa* polysaccharides enhance intestinal immunity (Yu et al., 2024). The botanical drug extract prepared from *C. officinalis* enhances immunity by increasing the NK cell activity and regulating the cytokine levels (Woo et al., 2019). *Salvia miltiorrhiza* polysaccharides have been proven to possess immune regulation and antioxidant abilities (Chen et al., 2017; Wang P et al., 2018). Another kidney-nourishing and blood-activating decoction containing similar Chinese plants (*C. chinensis* and *R. glutinosa*), has been shown to restore ovarian function and attenuate inflammation in a premature ovarian insufficiency mouse model (Wang T et al., 2018; Chen et al., 2021). Collectively, these outcomes suggest that BSHX decoction may improve fertility *via* mechanism involving immunomodulation and enhanced systemic stress resistance.

Heat stress is a major threat to reproductive function, disrupting proteostasis and accelerating cellular damage (Hsu et al., 2003). In *Caenorhabditis elegans* (*C. elegans*), the extracellular regulated kinase (ERK) mitogen-activated protein kinases (MAPK) pathway orchestrates a germline-to-soma signaling axis in response to heat stress, inducing the expression of secreted immune peptides and systemic stress responses (Ermolaeva et al., 2013). These findings point to a potential crosstalk between stress response and fertility regulation.

The mitochondrial unfolded protein response (UPR^mt^) is a crucial system stress response that maintains proteostasis. It has been implicated in both longevity and reproductive health (Su et al., 2010; Gómez-Valadés et al., 2021). Its activation has been demonstrated to enhance fertility and stress resistance in various models, including *Drosophila* and *in vitro* embryo systems (Baqri et al., 2014; Marei et al., 2019). However, the link between UPR^mt^ signaling and the fertility remains poorly understood.

*C. elegans* is a widely utilized alternative model in pharmacology, owing to its advantages such as rapid life cycle, high progeny output, and extensive mutant collections. It features a structurally simple yet fully developed reproductive system, along with a well-characterized oocyte development process. Oogenesis in nematodes exhibits considerable similarity to that in mammals. Previously, we found Bisphenol A (BPA), an environmental endocrine-disrupting chemical, significantly decreased brood size in *C. elegans* by increasing germline apoptosis and decreasing the number of diakinesis-stage oocytes per gonad arm (Wu et al., 2023). Notably, BSHX decoction effectively reversed BPA-induced reproductive damage, restoring brood size (Wu et al., 2023). Besides, *C. elegans* serves as a powerful model for studying innate immunity (Balla and Troemel, 2013; Liu et al., 2023). Multiple evolutionarily conserved signaling pathways, including the MAPK pathway, are known to mediate immune responses against pathogenic infection (Kumar et al., 2020). Herein, the present study aims to investigate the underlying mechanism by which BSHX decoction enhances fertility via modulating innate immunity and systemic stress resistance in *C. elegans*.

## 2 Materials and methods

### 2.1 Study design

This study employed a quantitative experimental design using *C. elegans* as an *in vivo* model to investigate the protective effects of BSHX decoction against BPA-induced mitochondrial dysfunction and heat stress.

### 2.2 *C. elegans* strains

The subsequent *C. elegans* strains were obtained from the *Caenorhabditis* Genetics Center (CGC), University of Minnesota, Minneapolis, MN, USA: Wild-type: N2.

Mutants and transgenic strains: ZD442 (agIs219 *atf-7(qd22)*), CB4037(*glp-1(e2141)*), BJS737 (*mpk-1(sbj10)*), KU25 (*pmk-1(km25)*), VC8 (*jnk-1(gk7)*), SA115 [*pie-1p::GFP::rho-1*+*unc-119(+)*], VC3201 (*atfs-1(gk3094)*), SJ4100 (zcIs13*[hsp-6::GFP]*), NL2099 (*rrf-3(pk1426)*), DCL569 (mkcSi13 [*sun-1p*::*rde-1*::*sun-1*+*unc-119*]), NR350 (kzIs20 [*hlh-1p*::*rde-1*+*sur-5p*::*NLS::GFP*), VP303 (kbIs7 [*nhx-2p*::*rde-1*+*rol-6*]), TU3401 (*myo-2p*::mCherry + *unc-119p*::*sid-1*], and PRJ112 (*pmk-1*::GFP + *rol-6*).

### 2.3 Reagents and materials

Bisphenol A (batch no.: B108652, purity ≥99%) was obtained from Aladdin Co., Ltd; TRIzol (R0016), mitochondrial membrane potential assay kit (C2006) were obtained from Beyotime Biotech Co., Ltd. A Mitochondrial Reactive Oxygen Species Assay Kit (40740ES50) and Hieff ^Ⓡ^ qPCR SYBR Green Master Mix (11201ES08) were obtained from Yeasen Biotech Co., Ltd.; Phanta^Ⓡ^ Max Super-Fidelity DNA Polymerase (P505-dl) was obtained from Vazyme Biotech Co., Ltd; PrimeScript™ RT Master Mix (TKR-RR036A) was obtained from Takara. Authentic standards of chlorogenic acid (batch no.: FY1435B516), loganin (batch no.: FY10B8023), paeoniflorin (batch no.: FY1589B310), verbascoside (batch no.: FY1624B0807), salvianolic acid B (batch no.: FY1167B8026), quercetin (batch no.: FY18B406), asperosaponin VI (batch no.: FY1136B1404), hyperoside (batch no.: FY154B313), and kaempferol (batch no.: FY67B803) were acquired from Feiyu Bio-Technology Limited Corporation, Nantong. The purity of all these metabolites was >98%. The seed of *C. chinensis* Lam. (batch no.: 210226), the radix of *R. glutinosa* (Gaertn.) Libosch. ex DC. (batch no.: 201228), the radix of *P. lactiflora* Pall. (batch no.: 210125), the radix of *P. veitchii* Lynch (batch no.: 201230), the fruit of *C. officinalis* Siebold & Zucc. (batch no.: 210108), the radix and rhizome of *S. miltiorrhiza* Bunge (batch no.: 210228), the radix of *D. asper* Wall. ex DC. (batch no.: 190125), and the carapax and plastron of *tortoise* (batch no.: 190867) were acquired from Tong Ren Tang pharmacy, Nanjing, China. All Chinese herbs were identified by Professor Sheng Guo from the Jiangsu Provincial Collaborative Innovation Center for Industrialization Process of Chinese Medicine Resources at Nanjing University of Chinese Medicine. All specimens are deposited at Nanjing University of Chinese Medicine.

### 2.4 Preparation of BSHX decoction

Eight traditional Chinese herbs, including the seed of *C. chinensis* Lam., the radix of *R. glutinosa* (Gaertn.) Libosch. ex DC., the radix of *P. lactiflora* Pall., the radix of *P. veitchii* Lynch, the fruit of *C. officinalis* Siebold & Zucc., the radix and rhizome of *S. miltiorrhiza* Bunge, the radix of *D. asper* Wall. ex DC., and the carapax and plastron of *tortoise*, were weighed equally (10 g each), soaked in 800 mL distilled water for 30 min. The first decoction was filtered, and these Chinese herbs were sequentially extracted with water twice for another 30 min. The filtrates were combined and vacuum-concentrated at 62 °C (EYELA rotavapor) to yield a final concentration of 0.5 g/mL, stored at 4 °C. Working solutions of 31.25, 62.5, and 125 mg/mL were prepared fresh before experiments.

### 2.5 *C. elegans* strain maintenance and treatment

Nematodes were cultivated on nematode growth medium (NGM) plates plated with *E. coli* OP50, following standard protocols (Brenner, 1974). Gravid adults were bleached to obtain age-synchronized L1 larvae. For exposure studies, L1 larvae were treated with 175 μg/mL Bisphenol A (BPA) (Sigma-Aldrich, St. Louis, MO, United States) or varying doses (31.25, 62.5, and 125 mg/mL) of BSHX decoction until day 3 adulthood at 20 °C. Treatments followed modified protocols based on Allard and Colaiácovo (2010). Experiments were conducted in triplicate.

### 2.6 Brood size assays

Fertility was assessed by brood size. Following BSHX treatment, brood size was measured by isolating ten individual nematodes per group. A single nematode was placed onto an NGM plate with OP50. Each day, all P0 nematodes were transferred to a new NGM plate, and progeny beyond the egg stage were counted to determine fertility (Wu et al., 2023).

### 2.7 Heat stress assays

Nematodes were seeded on 60 mm NGM agar plates (50 nematodes/plate) and incubated at 35 °C for heat stress. Survival was recorded every 1–2 h until all nematodes perished (Ruan et al., 2016).

### 2.8 Gonads separation

Nematodes were dissected in a drop of M9 buffer by severing at the pharynx-intestine junction using 1 mL syringe needles. Gonads were released and transferred into sterile 1.5 mL tubes containing clean buffer. Approximately 50 gonads were harvested per group for RNA analysis.

### 2.9 Fluorescence microscopy

Post-treatment nematodes (Day 2 adults from strains ZD442, SA115, and SJ4100) were mounted on agar pads, paralyzed with 50 mM levamisole, and imaged under a Zeiss Scope A1 fluorescence microscope (Carl Zeiss AG, Jena, Germany). Fluorescence intensities of p38–ATF-7-regulated immunity protein (T24B8.5), RhoGTPase (RHO-1), heat shock protein 6 (HSP-6), were quantified using ZEN software (Zeiss, Oberkochen, Germany).

### 2.10 Mitochondrial reactive oxygen species and membrane potential assays

Mitochondrial reactive oxygen species (Mito-ROS) was assessed using MitoTracker Red CM-H2XRos (Yeasen Biotechnology, Shanghai, China); mitochondrial membrane potential was analyzed with JC-1 dye (Invitrogen, Waltham, MA, USA). Nematodes from each experimental group were collected into centrifuge tubes, washed 2–3 times with M9 solution, and the supernatant was discarded. Then, 100 μL of 50 μM Mito-ROS or JC-1 working fluids was added. For Mito-ROS staining, samples were incubated at 37 °C for 1.5 h. For JC-1 staining, samples were incubated in a 20 °C shaker for 2 h. After staining, washing nematodes 2–3 times with M9 solution and transferring them to agarose pads for observation under Zeiss fluorescence microscope (Xiao et al., 2025).

### 2.11 RNA interference

RNA interference (RNAi) by feeding was performed using the *E. coli strain* HT115 carrying *hsp-6/T24B8.5* RNAi constructs. Strains DCL569, NR350, VP303, and TU3401 are sensitive to RNAi limited to the germline, muscle, intestine, and neurons, respectively. Following RNAi of *hsp-6/T24B8.5*, nematodes were fed the *E*. *coli* strain HT115. L1 larvae were incubated on plates containing RNAi bacteria or the control vector at 15 °C for 36 h, then at 23 °C for 36 h. *E*. *coli* strain HT115 carrying the empty RNAi vector L4440 was used as a control (Xiao et al., 2025). RNAi primers are listed in Supplementary Table S1.

### 2.12 RNA extraction and qRT-PCR

Total RNA was extracted from Day 2 adults nematodes using Beyozol reagent (Beyotime Institute of Biotechnology, Haimen, China) and reverse-transcribed into cDNA with PrimeScript™ RT Master Mix (Takara Bio, Inc., Shiga, Japan). qPCR was performed using LightCycler® SYBR Green I (Roche Life Science, Penzberg, Germany), with *act-3* as the internal control (Ruan et al., 2016). The primers used are listed in Supplementary Table S1.

### 2.13 LC/Q-TOF-MS analysis of BSHX metabolites

The BSHX decoction prepared according to Method 2.4 was analyzed using an Agilent 6546 LC/Q-TOF mass spectrometry (Agilent Technologies, USA) for metabolites profiling. The stock solution of BSHX was diluted 5-fold with methanol, followed by high-speed centrifugation. The supernatant was collected for injection analysis. Chromatographic separation was performed on an Agilent Zorbax SB-C18 column (2.1 × 150 mm, 3.5 μm) at 35 °C using a gradient mobile phase containing 0.025% formic acid in water (solvent A) and methanol (solvent B). The linear gradients were as follows: 5% B for 0–3 min, 5%–20% B for 3–16 min, 20%–50% B for 16–24 min, 50%–95% B for 24–32 min, 95% B for 32–34 min, 95%–5% B for 34–35 min, and 5% B for 35–40 min. The mobile phase flow rate was 0.2 mL/min, and the injection volume was 2 μL. Mass spectrometry parameters were set as follows: a negative DuoSpray ion source was used, ion source gas 1,40 psi; ion source gas 2, 45 psi; curtain gas, 40 psi, temperature, 550 °C; IonSpray voltage, −4500 V; declustering potential (DP), 100 V; collision energy (CE) at −10 V and −35 V for MS1 and MS2, respectively, with a CE spread of 20 V for MS2. Full scan mass spectra were acquired in negative ion mode over a mass range of m/z 100–1,500.

### 2.14 Bioinformatics analysis of seven metabolites from the Bushen Huoxue decoction for human hepatotoxicity

Identification of metabolite targets: The SMILES structures of the seven metabolites (loganin, paeoniflorin, verbascoside, salvianolic acid B, quercetin, asperosaponin VI, hyperoside) identified via LC-Q-TOF-MS were obtained from the PubChem database. These SMILES strings were subsequently input into the SwissTargetPrediction platform (http://www.swisstargetprediction.ch/) to retrieve their respective target information.

Collection of hepatotoxicity-related targets: Potential hepatotoxicity targets were retrieved from the GeneCards (https://www.genecards.org/) and OMIM (https://www.omim.org/) databases using the keyword “human hepatotoxicity”. All collected targets were merged, and duplicate entries were removed using the ggvenn package in R software (version 4.4.0).

Identification of common targets: The ggvenn package in R software (version 4.4.0) was used to analyze the intersection between the target genes of the seven metabolites and the hepatotoxicity-related genes. This analysis generated result files detailing the intersection genes, network relationships, and node attributes, thereby establishing the set of potential targets through which the metabolites might induce hepatotoxicity.

GO and KEGG enrichment analysis: Gene Ontology (GO) annotation and KEGG pathway enrichment analyses were performed on the intersection gene set using R software (version 4.4.0) and packages including colorspace, stringi, ggplot2, circlize, RColorBrewer, and ggpubr. Bar charts and bubble charts were plotted to visualize the results. The fundamental data for Biological Process (BP), Molecular Function (MF), Cellular Component (CC), and KEGG pathways were obtained. For visualization, the top 10 GO terms and the top 20 KEGG pathways were selected.

### 2.15 Statistical analysis

All data are reported as mean ± SEM. SPSS 12.0 (IBM Corp., NY, USA) was utilized to conduct statistical analyses. Group differences were evaluated via one-way ANOVA followed by Dunnett’s *post hoc test* or independent *t*-tests. Survival data were evaluated using the log-rank (Mantel-Cox) test. A p-value ≤0.05 was considered statistically significant.

## 3 Results

### 3.1 BSHX decoction enhances heat-stress resistance by stimulating the MAPK pathway in the gonads

**FIGURE 1:**
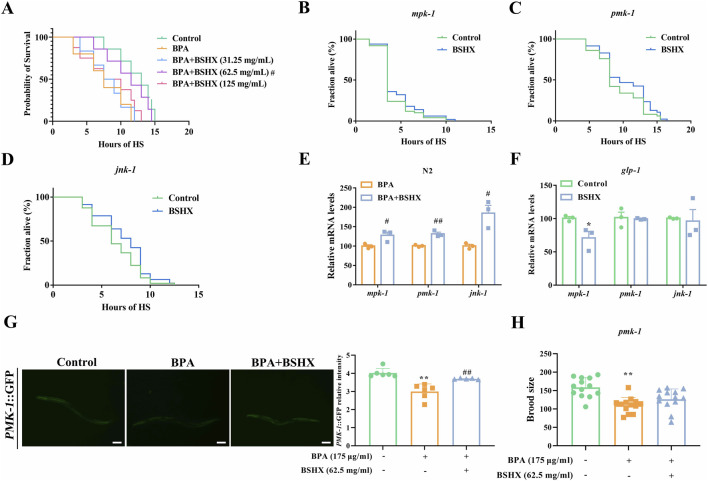
BSHX decoction improves heat-stress resistance by activating the MAPK signaling pathway in the gonads. **(A)** Treatment with BSHX at 62.5 mg/mL significantly increased the survival rate compared with BPA-exposed nematodes. **(B,D)** BSHX failed to improve the heat-stress survival rate in the *mpk-1* **(B)**, *pmk-1* **(C)**, and *jnk-1* **(D)** mutants. **(E)** BSHX upregulated the transcriptional levels of all three genes in BPA-exposed nematodes. **(F)** BSHX failed to upregulate the transcriptional levels of *pmk-1* and *jnk-1* in *glp-1* mutants. **(G)** Left: Representative images of PMK-1:GFP expression in PRJ112 nematodes treated with BPA and BSHX (scale bar, 100 μm). Right: BSHX reversed the BPA-induced fluorescence intensity of PMK-1:GFP in PRJ112 nematodes. **(H)** BSHX failed to reverse BPA-induced reproductive damage in *pmk-1* mutants. All data are presented as mean ± SEM. #*P* < 0.05 vs. BPA group. ##*P* < 0.01 vs. BPA group. *P < 0.05 vs. control group. **P < 0.01 vs. control group. BSHX, BuShen HouXue; MARK, mitogen activated protein kinase; BPA, Bisphenol A.

Previous findings indicate that 62.5 mg/mL BSHX decoction ameliorates BPA-induced reproductive damage (Wu et al., 2023). We first assessed its effect on heat stress resistance. Treatment with BSHX at concentrations of 32.25, 62.5, and 125 mg/mL revealed that 62.5 mg/mL significantly enhanced the heat-stress resistance in BPA-exposed nematodes (Figure 1A). Given the established role of mitogen-activated protein kinase (MAPK) signaling pathway in promoting proteostasis and systemic stress resistance (Ermolaeva et al., 2013), we next investigated its involvement in BSHX-mediated protection. In *C. elegans*, *mpk-1*, *pmk-1*, and *jnk-1* are downstream genes of the extracellular signal-regulated kinase MAPK (ERK MAPK), p38 MAPK (PMK MAPK), and c-Jun N-terminal kinase (JNK MAPK) signaling pathways, respectively. Consequently, we conducted heat stress assays using *mpk-1*, *pmk-1*, and *jnk-1* loss-of-function mutants. The concentration of 62.5 mg/mL BSHX decoction was used in the following experiments.

As shown in Figures 1B–D, BSHX failed to improve the heat-stress survival rate in the *mpk-1*, *pmk-1*, and *jnk-1* mutants, indicating that all three MAPK genes are involved in the protective effect. Consistent with this, BSHX treatment upregulated the transcriptional levels of all three genes in BPA-exposed nematodes (Figure 1E). However, this upregulation of *pmk-1* and *jnk-1* were absent in *glp-1* mutants with a germline-less phenotype (Figure 1F), suggesting a germline-dependent mechanism. Furthermore, increased fluorescence intensity of PMK-1:GFP in BPA-exposed PRJ112 nematodes following BSHX treatment confirmed that BSHX enhances PMK-1 protein level (Figure 1G). Given the known role of *pmk-1* in germline apoptosis (Pei B et al., 2008), we further demonstrated that BSHX failed to improve the brood size in *pmk-1* mutants or in nematodes subjected to *pmk-1* RNAi under BPA exposure. (Figure 1H; Supplementary Figure 1A). In summary, BSHX enhances heat stress resistance via upregulating *pmk-1* and *jnk-1* expression in the gonads.

### 3.2 BSHX decoction increases fertility by upregulating the transcriptional levels of PMK-1–regulated immune peptides

**FIGURE 2:**
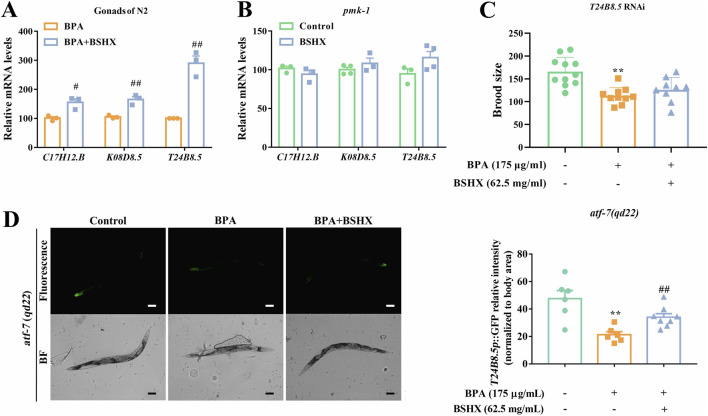
BSHX decoction improves fertility by improving the transcriptional levels of PMK-1–target immune peptides. **(A)** BSHX elevated the transcription of immune peptide genes in the gonads of BPA-exposed N2 nematodes. **(B)** BSHX failed to upregulate the immune peptide gene expression in *pmk-1*mutants. **(C)** BSHX failed to rescue BPA-induced reproductive damage in *T24B8.5* RNAi nematodes. **(D)** Left: Representative images of T24B8.5:GFP expression in *atf-7(qd22)* nematodes treated with BPA and BSHX (scale bar, 100 μm). Right: BSHX reversed the BPA-induced fluorescence intensity of T24B8.5:GFP in *atf-7(qd22)*. All data are presented as mean ± SEM. #*P* < 0.05 vs. BPA group. ##*P* < 0.01 vs. BPA group. *P < 0.05 vs. control group. **P < 0.01 vs. control group. BSHX, BuShen HouXue; MARK, mitogen activated protein kinase; BPA, Bisphenol A.

Innate immune signaling is known to mediate systemic responses that enhance survival (Karpac et al., 2011). A key regulator of this process is the conserved PMK-1 MAPK pathway, which controls both basal and pathogen-induced expression of immune effectors (Shivers et al., 2010). *C17H12.8*, *K08D8.5*, and *T24B8.5* have been identified as putative secreted immune peptides in nematodes (Troemel et al., 2006; Shivers et al., 2010). Herein, BSHX significantly upregulated the transcriptional levels of *C17H12.8*, *K08D8.5*, and *T24B8.5* in BPA-exposed nematodes and in isolated nematode gonads (Figure 2A; Supplementary Figure 1B). The decoction failed to regulate the expression levels of any of these three genes in the *pmk-1* mutant (Figure 2B). Furthermore, the brood size in BSHX-treated *T24B8.5 RNAi* nematodes was not significantly different from that in the BPA group (Figure 2C).

The *atf-7* transcriptional reporter strain, which contains the *T24B8.5* promoter (a PMK-1–regulated gene) fused to green fluorescent protein (GFP), serves as an *in vivo* sensor of PMK-1 MAPK pathway activity. Here, we used *atf-7 (qd22)* nematodes to observe the effects of BSHX on T24B8.5 protein expression. BPA decreased the fluorescence intensity of T24B8.5:GFP in *atf-7(qd22)* nematodes, whereas BSHX reversed this BPA-induced reduction (Figure 2D). Combined with our previous finding that 62.5 mg/mL BSHX significantly restored fertility in BPA-exposed nematodes (Wu et al., 2023), these results indicate that BSHX enhances fertility by upregulates the immune factor *T24B8.5* through the PMK-1 signaling pathway.

### 3.3 BSHX improves fertility via UPR^mt^-mediated protein homeostasis

**FIGURE 3:**
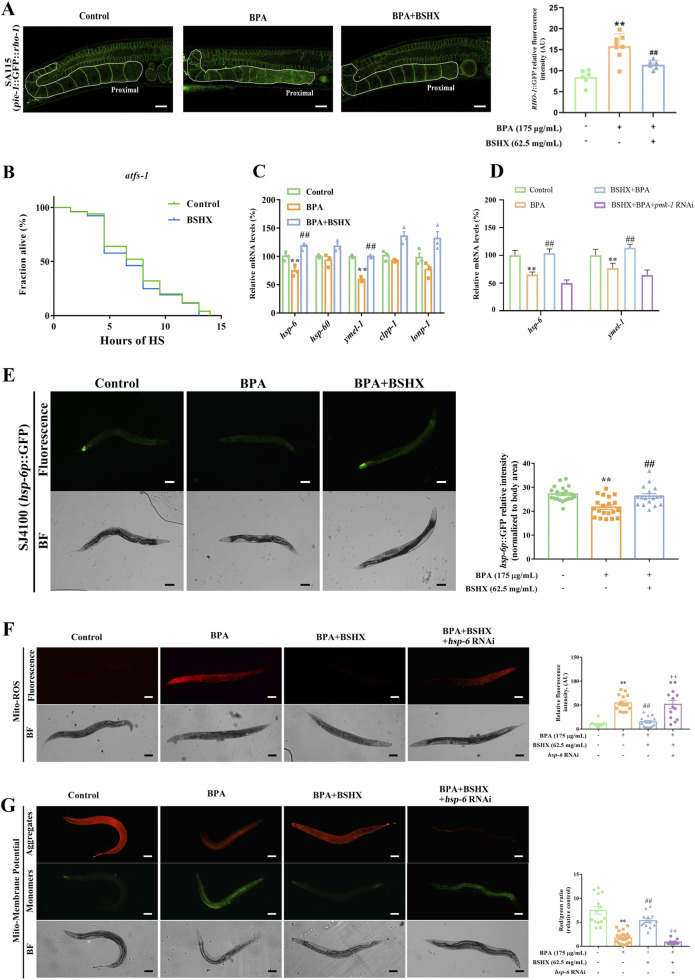
BSHX decoction enhances fertility by activating the UPR^mt^ via *hsp-6*. **(A)** Left: BSHX effectively reversed the BPA-induced protein accumulation (aggregation-prone protein RHO-1:GFP) in the proximal gonad of the SA115 nematodes; white frames indicate the gonad arm region analyzed (scale bar: 20 μm). Right: Quantification of relative RHO-1:GFP fluorescence intensity, reflecting protein aggregation levels. **(B)** BSHX failed to improve the heat-stress survival rate in the *atfs-1* mutants, indicating dependence on UPR^mt^ signaling. **(C)** BSHX upregulated the transcriptional levels of UPR^mt^-related genes in BPA-exposed nematodes. **(D)** BSHX failed to upregulate the UPR^mt^-related gene expression in *pmk-1* RNAi nemataodes, highlighting the regulatory cross-talk between MAPK and mitochondrial stress pathways. **(E)** Left: BSHX reversed the BPA-induced fluorescence intensity of HSP-6:GFP in SJ4100 nematodes (scale bar: 100 μm). Right: Corresponding quantification of HSP-6:GFP signal intensity, illustrating activation of mitochondrial chaperone pathways. **(F,G)** BSHX reversed the BPA-induced mitochondrial function impairment, while *HSP-6* RNAi abolished the beneficial effect of BSHX on mitochondria **(F)** Mitochondrial reactive oxygen species (ROS) levels; **(G)** Mitochondrial membrane potential (ΔΨm), with left panels showing representative fluorescence images (scale bars: 100 μm) and right panels displaying quantification of fluorescence intensity. All data are presented as mean ± SEM. **P < 0.01 vs. control group. ##*P* < 0.01 vs. BPA group. ^++^ *P* < 0.01 vs. BSHX group. BSHX, BuShen HouXue; BPA, Bisphenol A.

Disruption of protein homeostasis is a hallmark of aging and degenerative diseases, characterized by the accumulation and impaired clearance of abnormal proteins. In *C. elegans*, RHO-1 protein becomes insoluble in oocytes along with aging (David et al., 2010). GFP-tagged RHO-1, an aggregation-prone protein, forms stationary aggregates restricted to proximal oocytes, establishing it as a validated marker for monitoring damage clearance in this compartment (Bohnert and Kenyon, 2017). Using the SA115 transgenic strain, which exhibits oocyte-specific RHO-1:GFP fluorescence, we found that BPA significantly promoted the accumulation of RHO-1 in proximal oocytes per gonad arm. Importantly, BSHX effectively reversed this BPA-induced protein accumulation (Figure 3A).

The UPR^mt^ is a conserved signaling pathway that maintains mitochondrial proteostasis and mitigates abnormal protein aggregation, serving as an important cytoprotective mechanism. Activation of UPR^mt^ triggers the nuclear translocation of the transcription factor activating transcription factor 1 (ATFS-1), which induces the expression of downstream genes (*hsp-6*, *hsp-60*, *ymel-1*, *clpp-1*, and *lonp-1*), thereby restoring mitochondrial protein homeostasis. To investigate whether BSHX activates UPR^mt^, we first used the VC3201 strain (*atfs-1* loss-of-function mutant). As shown in Figure 3B, BSHX failed to improve the heat stress survival rate in VC3201 nematodes, indicating a dependency on ATFS-1.

We next examined the transcriptional levels of UPR^mt^ markers. qRT-PCR analysis revealed that BPA significantly downregulated the transcriptional levels of *hsp-6* and *ymel-1*, whereas BSHX restored their expression (Figure 3C). Consistent with this, in the SJ4100 transgenic strain, which carries an HSP-6:GFP reporter with fluorescence localized predominantly in the caudal intestine, BPA markedly reduced GFP intensity, and this reduction was reversed by BSHX (Figure 3E).

To determine whether the PMK-1 MAPK pathway is involved in UPR^mt^ activation, we detected the transcriptional levels of *hsp-6* and *ymel-1* in PMK-1 RNAi nematodes. Knockdown of PMK-1 significantly suppressed the BSHX-induced upregulation of both genes (Figure 3D), suggesting that PMK-1 acts upstream of UPR^mt^

We further assessed mitochondrial function. BPA significantly elevated mitochondrial-ROS and reduced mitochondrial membrane potential, both of which were partially rescued by BSHX (Figures 3F,G). Importantly, *HSP-6* RNAi abolished the beneficial effect of BSHX on these mitochondrial parameters (Figures 3F,G). Together, these results suggest that UPR^mt^ activation contributes to BSHX-induced heat-stress resistance, and that BSHX enhances thermotolerance through an *hsp-6*-mediated recovery of mitochondrial function.

### 3.4 BSHX enhances fertility via a multi-tissue UPR^mt^ response

The UPR^mt^ can coordinate stress resistance across different tissues, exerting a synergistic effect on organismal survival (Durieux et al., 2011). The UPR^mt^-regulated gene *hsp-6* serves as a key indicator of this response. To investigate whether BSHX acts through UPR^mt^ activation in multiple tissues, we employed tissue-specific RNAi strains targeting the germline (DCL569), intestine (VP303), muscle (NR350), and neurons (TU3401).

**FIGURE 4:**
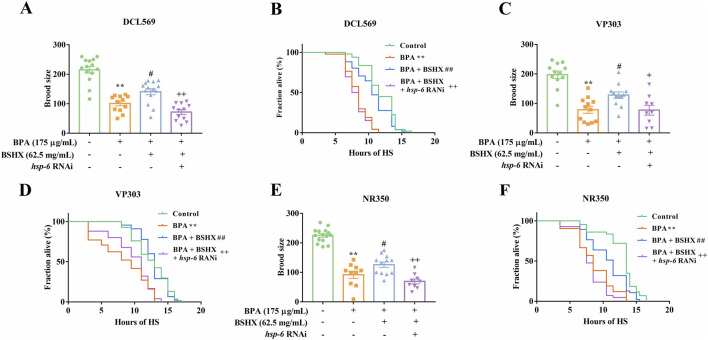
BSHX decoction enhances fertility through tissue-specific activation of the UPR^mt^. BPA reduced both brood size and heat-stress resistance across all tissue-specific RNAi strains. Tissue-specific *hsp-6* knockdown was used to dissect the contributions of UPR^mt^ in germline, intestine, and muscle to the fertility-enhancing effects of BSHX in BPA-exposed nematodes. **(A,B)** Germline-specific RNAi strain DCL560: **(A)** Brood size and **(B)** survival following heat stress, showing partial attenuation of BSHX effects upon *hsp-6* suppression in the germline. **(C,D)** Intestine-specific RNAi strain VP303: **(C)** Brood size and **(D)** heat-stress resistance, indicating a significant role for intestinal UPR^mt^ in mediating BSHX-driven protection. **(E,F)** Muscle-specific RNAi strain NR350: **(E)** Brood size and **(F)** survival under heat stress, revealing the contribution of muscle-localized *hsp-6* activity to BSHX-mediated reproductive and stress resilience benefits. All data are presented as mean ± SEM. **P < 0.01 vs. control group. #*P* < 0.05 vs. BPA group. ##*P* < 0.01 vs. BPA group. ^+^ *P* < 0.05 vs. BSHX group. ^++^ *P* < 0.01 vs. BSHX group. BSHX, BuShen HouXue; BPA, Bisphenol A; HS, Heat stress.

BPA reduced both brood size and heat-stress resistance across these strains (Figure 4), while BSHX effectively reversed BPA-induced deficits, restoring fertility and heat-stress resistance in the gonadal (Figures 4A,B), intestinal (Figures 4C,D), and muscle-specific (Figures 4E,F) RNAi backgrounds, as well as in neurons (Supplementary Figure 2). To determine whether these improvements depended on *hsp-6*, we performed HSP-6 RNAi in each strain. HSP-6 RNAi inhibited the beneficial effects of BSHX on brood size of stress resistance in the germline, intestine, and muscle (Figure 4). In contrast, HSP-6 RNAi had no significant effect in neurons (Supplementary Figure 2). Thus, these results demonstrate that BSHX enhances fertility and stress resistance in an HSP-6-dependent manner*,* and that this mechanism operates synergistically across gonadal, intestinal, and muscular tissues.

### 3.5 A combination of metabolites identified from the BSHX decoction significantly enhances heat-stress resistance and fertility by activating the PMK-regulated innate immune peptide T24B8.5

**FIGURE 5:**
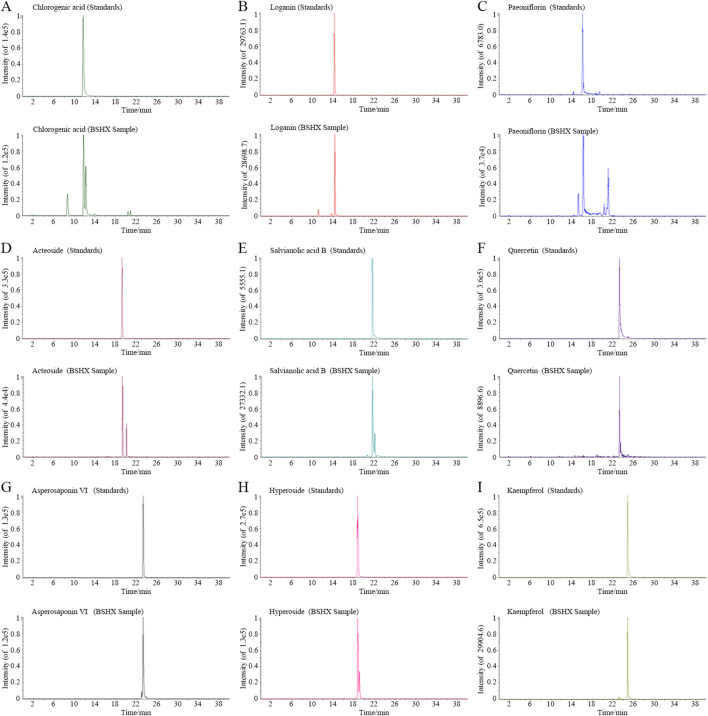
Representative active metabolites in BSHX decoction identified by extracted ion chromatograms (EICs). **(A–I)** EICs of nine bioactive metabolites detected in BSHX decoction, compared to corresponding reference standards: **(A)** Chlorogenic acid, **(B)** Loganin, **(C)** Paeoniflorin, **(D)** verbascoside, **(E)** Sal B, **(F)** Quercetin, **(G)** Asp VI, **(H)** Hyperoside, and **(I)** Kaempferol. Each panel shows the standard chromatogram (upper panel) and the corresponding metabolite peak in BSHX decoction (lower panel), confirming metabolite identity and presence in the extract. BSHX, BuShen HouXue.

Our previous study detected paeoniflorin, loganin, salvianolic acid B (Sal B), asperosaponin VI (Asp VI), verbascoside, catalpol, and quercetin (Que) in the BSHX decoction (Wu et al., 2023). Here, we expanded the metabolite profiling using LC-Q-TOF, which confirmed the presence of chlorogenic acid, loganin, paeoniflorin, verbascoside, Sal B, quercetin, Asp VI, hyperoside, and kaempferol (Figure 5). Notably, Sal B, Asp VI, and quercetin were previously found to be absorbed by nematodes following BSHX treatment (Wu et al., 2023), suggesting their potential contribution to the improved fertility. To test this, we administered these three metabolites in combination at low, individually ineffective doses.

**FIGURE 6:**
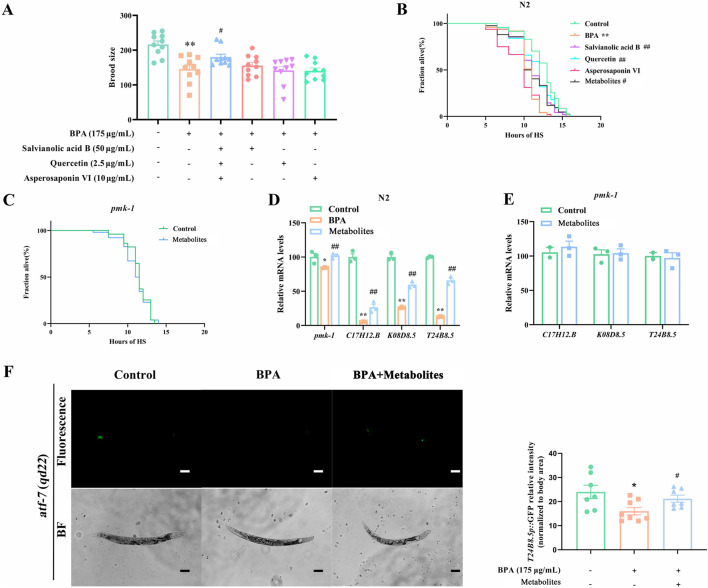
Combined metabolites enhance fertility and heat-stress resistance *via* PMK-1-targeted the innate immune effector T24B8.5. **(A)** A combination of metabolites (Sal B, Quercetin, Asp VI) significantly recovered the brood size in BPA-exposed nematodes. **(B,C)** The combination of metabolites improved the heat-stress survival rate in the BPA-exposed nematodes **(B)**, while *pmk-1* knockdown abolished the beneficial effect of BSHX on heat resistance **(C)**, revealing dependency on PMK-1 signaling for stress resistance. **(D)** The combination of metabolites upregulated the transcriptional levels of *pmk-1* and immune peptide genes in BPA-exposed nematodes. **(E)** The combination of metabolites failed to upregulate the transcriptional levels of immune peptide genes in *pmk-1* mutants. **(F)** Left: The combination of metabolites reversed the BPA-induced fluorescence intensity of T24B8.5:GFP in *atf-7(qd22)* (scale bar: 100 μm). Right: Quantification of T24B8.5:GFP signal intensity. Data are reported as mean ± SEM. *P < 0.05 vs. control group. **P < 0.01 vs. control group. #*P* < 0.05 vs. BPA group. ##*P* < 0.01 vs. BPA group. BPA, Bisphenol **(A)**. BF, Bright field.

We first screened various ratios of Sal B (30–60 μg/mL), quercetin (0.5–25 μg/mL), and Asp VI (5–20 μg/mL) for their effects on fertility (Supplementary Figure 3). A specific combination (Sal B 50 μg/mL + Que 2.5 μg/mL + Asp VI 10 μg/mL) significantly enhanced the brood size of BPA-exposed nematodes, whereas none of the individual metabolites showed this effect (Figure 6A). The same combination also significantly increased the heat stress survival of BPA-exposed nematodes (Figure 6B), indicating that the metabolite combination (Sal B + Que + Asp VI) significantly rescues both fertility and heat-stress resistance.

The metabolite combination failed to elevate the heat-stress resistance of the *pmk-1* mutant (Figure 6C). The combination significantly upregulated the transcriptional levels of *pmk-1* and immune genes *C17H12.8*, *K08D8.5*, and *T24B8.5* in BPA-exposed nematodes, but had no effect on these immune genes in the *pmk-1* mutant (Figures 6D,E). Furthermore, the combination significantly increased the fluorescence intensity of T24B8.5 in the *atf-7(qd22)* nematodes under BPA exposure (Figure 6F). Collectively, these results suggest the metabolite combination increases immunity response and stress resistance by upregulating the immune peptides T24B8.5 via the PMK-1 MAPK pathway.

### 3.6 The metabolite combination improves fertility by activating the UPR^mt^ across multiple tissues

**FIGURE 7:**
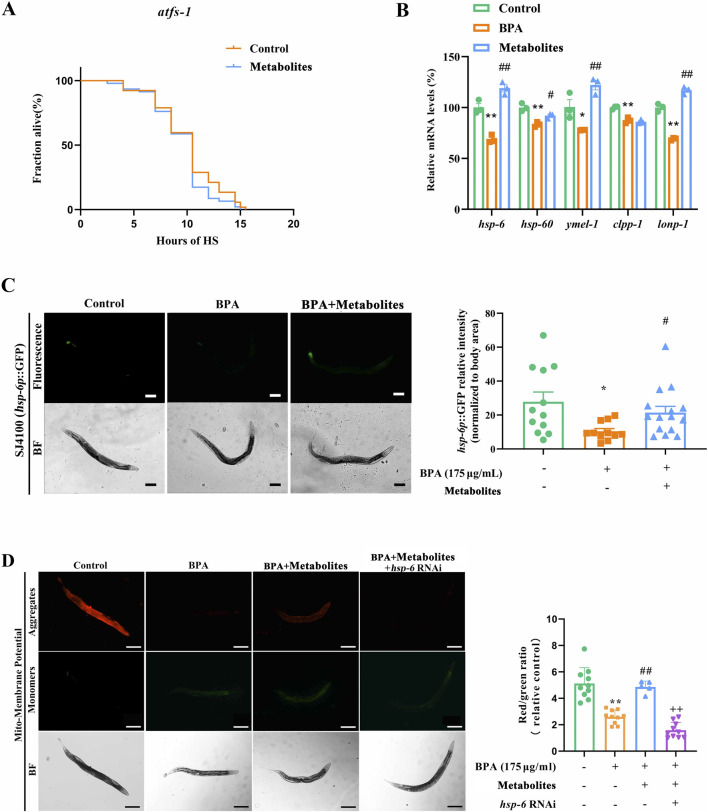
Combined metabolites restore fertility by activating *hsp-6*-mediated UPR^mt^. **(A)** The combination of metabolites failed to improve the heat-stress survival rate in the *atfs-1* mutants, indicating UPR^mt^ dependence for enhanced stress tolerance. **(B)** The combination of metabolites upregulated the transcriptional levels of UPR^mt^-related genes in BPA-exposed nematodes, demonstrating transcriptional activation of mitochondrial stress pathways. **(C)** Left: The combination of metabolites reversed the BPA-induced fluorescence intensity of HSP-6:GFP in SJ4100 nematodes (scale bar: 100 μm). Right: Quantification of HSP-6:GFP fluorescence intensity, confirming UPR^mt^ induction by metabolites. **(D)** Left: The combination of metabolites reversed the BPA-induced mitochondrial membrane potential (ΔΨm), while *HSP-6* RNAi abolished the beneficial effect of BSHX on mitochondrial membrane potential (ΔΨm) (scale bars: 100 μm), Right: Corresponding histogram quantifying ΔΨm signal intensity, revealing functional mitochondrial restoration via *hsp-6*. Data are reported as mean ± SEM. *P < 0.05 vs. control group. **P < 0.01 vs. control group. #*P* < 0.05 vs. BPA group. ##*P* < 0.01 vs. BPA group. BPA, Bisphenol A. BF, Bright field.

Firstly, we observed that the combination failed to improve heat stress survival in *atfs-1* mutants (Figure 7A). The combination significantly upregulated the transcriptional levels of *hsp-6, hsp-60, ymel-1*, and *lonp-1*, increased the fluorescence intensity of HSP-6:GFP, and increased the mitochondrial membrane potential in BPA-exposed nematodes (Figures 7B–D). Furthermore, *hsp-6* RNAi reversed the beneficial effect of the metabolite combination on the mitochondrial membrane potential (Figure 7D).

**FIGURE 8:**
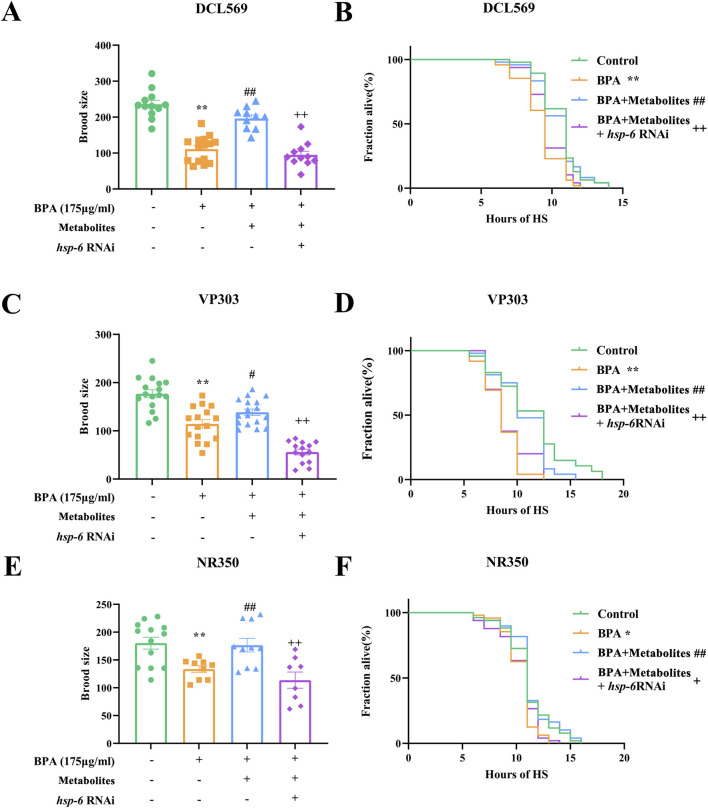
Combined metabolites enhance fertility through tissue-specific *hsp-6*-mediated mitochondrial UPR activation. Tissue-specific RNAi strains were used to evaluate the role of *hsp-6* in germline, intestinal, and muscle tissues in mediating the fertility and stress resistance effects of metabolite treatment under BPA-induced stress. **(A,B)** DCL560 (germline-specific RNAi): **(A)** Brood size and **(B)** survival following heat stress, demonstrating partial attenuation of metabolite-induced benefits upon *hsp-6* knockdown in the germline. **(C,D)** VP303(intestine-specific RNAi): **(C)** Brood size and **(D)** heat-stress survival, highlighting a critical role for intestinal hsp-6 in mediating metabolite-driven resilience. **(E,F)** NR350 (muscle-specific RNAi): **(E)** Brood size and **(F)** survival post heat stress, indicating that muscle-localized mitochondrial UPR also contributes to improved reproductive and stress outcomes.

Next, the inter-tissue synergistic effect of the metabolite combination was detected. BPA exposure decreased brood size and shortened the survival under heat-stress (Figure 8), while the combination counteracted these effects, increasing both brood size and survival in DCL569 (Figures 8A,B), VP303 (Figures 8C,D), and NR350 nematodes (Figures 8E,F). Importantly, HSP-6 RNAi suppressed these beneficial effects in all three strains. Together, these results suggest that the metabolite combination improves fertility through a coordinated inter-tissue UPR^mt^.

### 3.7 Functional enrichment analysis of common target genes between the seven metabolites in the decoction and human hepatotoxicity

**FIGURE 9:**
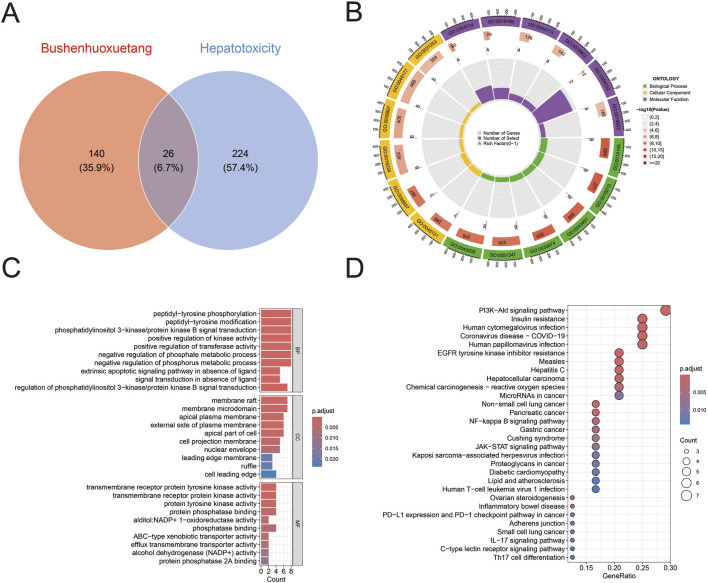
Network analysis of detected metabolites in hepatotoxicity. **(A)** Venn diagram representing the intersection of targets associated with metabolites and hepatotoxicity-associated targets. **(B,C)** Gene ontology enrichment results across biological process, cellular metabolite, and molecular function categories, illustrated by bubble chart **(B)** and bar chart **(C)**. **(D)** Significantly enriched pathways identified through Kyoto Encyclopedia of Genes and Genomes analysis.

Firstly, we screened the common target genes between the seven metabolites in the decoction and human hepatotoxicity. The seven metabolites of Bushen Huoxue decoction yielded a total of 140 potential targets. By searching the GeneCards and OMIM databases with the keyword “human hepatotoxicity,” 224 related target genes were obtained. Intersection analysis revealed 26 common genes (Figure 9A): *BCL2L1*, *F2*, *PTPA*, *STAT3*, *XDH*, *EGFR*, *GSK3B*, *KDR*, *ALK*, *ABCB1*, *ABCG2*, *AKR1C3*, *AKR1A1*, *INSR*, *ACHE*, *SLC22A12*, *CDK6*, *AHR*, *PARP1*, *TOP1*, *TERT*, *PTPN1*, *ADAM17*, *PTGS2*, *TNF*, and *IL2*.

As the liver is an organ susceptible to adverse drug effects, we investigated the potential signaling pathways through which the aforementioned metabolites may cause hepatotoxicity. GO and KEGG enrichment analyses were conducted using R software. GO analysis revealed that the common targets were significantly enriched in biological processes (BP) including protein phosphorylation, phosphatidylinositol 3-kinase/protein kinase B signal transduction, and positive regulation of kinase and transferase activity. In the cellular component (CC) category, these targets were primarily localized to membrane rafts, membrane microdomains, and the apical plasma membrane. For molecular function (MF), the most significantly enriched terms were protein kinase activity and phosphatase binding (Figures 9B,C). KEGG pathway analysis further demonstrated that these targets are predominantly involved in the PI3K-Akt signaling pathway (Figure 9D).

## 4 Discussion

This study illustrates a novel mechanism by which the BSHX decoction enhances fertility by modulating systemic stress responses in *C. elegans*. We demonstrate that BSHX activates the conserved PMK-1 and JNK-1 MAPK signaling pathways in germ cells, which subsequently induces systemic stress resistance. Notably, PMK-1 activation upregulates the immune peptide T24B8.5 and initiates the UPR^mt^, thereby improving proteostasis and oocyte quality. Together, these coordinated responses in germline and somatic tissues ultimately enhance fertility, supporting our hypothesis that BSHX enhances fertility by elevating systemic resilience.

Our findings are consistent with previous studies demonstrating the pivotal role of PMK-1MAPK pathway in mediating immune defense and stress responses in *C. elegans* (Kumar et al., 2020). The innate immune response in nematodes is regulated through the secretion of antimicrobial peptides (Ermolaeva et al., 2013). Specifically, T24B8.5 has been identified as a key immune peptide (Troemel et al., 2006; Shivers et al., 2010). In line with this, we observed strong induction of immune-related factors in germlines isolated from BSHX-treated nematodes (Figure 2A). Moreover, BSHX significant elevated T24B8.5 expression even in *atf-7* nematodes (Figure 2D). These outcomes indicate that germline PMK-1 activation by BSHX results in the induction of immune peptides such as T24B8.5.

We further show that the activation of PMK-1 induces the UPR^mt^ response (Figure 3D). A decline in mitochondrial stress response has been linked to the accumulation of damaged proteins and elevated reactive oxygen species (ROS) during aging, positioning UPR^mt^ as a key mechanism in anti-aging research (Seli et al., 2019). Its role in reproductive aging is also emerging, with genes such as *clpp-1* and *hsp-6* implicated in maintaining oocyte function and mitochondrial integrity (Wang X et al., 2018; Ergun et al., 2024; Zhou et al., 2024). Extending these observations, we found that BSHX enhances mitochondrial membrane potential and reduces Mito-ROS levels by upregulating *hsp-6* expression, and enhances fertility and heat-stress resistance through *hsp-6* in both germline and intestinal tissues, supporting a model of inter-tissue signaling (Figures 3, 4). Therefore, BSHX increases oocyte development through a *hsp-6*-mediated UPR^mt^.

Recent studies highlight that intestinal dysfunction can trigger immune-inflammatory responses, leading to reproductive diseases via a “gut-gonadal axis”. Metabolites or drugs such as curcumin, paeoniflorin, or the combination of metformin and curcumin have shown efficacy in treating female reproductive diseases by maintaining intestinal barrier function and reducing chronic inflammation (Feghhi et al., 2024; Song et al., 2024; Yang et al., 2024). In this context, we found that BSHX enhances fertility via the activation of UPR^mt^ and the heat-shock signaling pathway (another major anti-stress response) in the intestine (Figures 3, 4) (Wu et al., 2023). BSHX also decreased intestinal ROS and ameliorated intestinal permeability, mediated in part by the heat stress response (Wu et al., 2023). As a central immune organ in *C. elegans,* the intestine produces neurotransmitters, secretin, and various immune peptides. We propose that in nematodes, the intestine communicates with the gonad via the pseudocoelomic tract, analogous to the mammalian circulatory system. For example, the yolk proteins are secreted from the intestine into the pseudocoelomic tract as free-floating granules before being transported to the gonad (Kimble and Sharrock, 1983). Therefore, we hypothesize that BSHX facilitates the secretion of certain factors from the intestine that reach the gonad through the pseudocoelomic tract, in a manner highly dependent on the UPR^mt^ and heat stress response pathways.

Traditional Chinese medicine decoctions are complex multi-metabolite systems, whose remarkable therapeutic effects likely arise from the synergistic actions of numerous active metabolites on multiple targets and signaling pathways. In the metabolite analysis of the decoction, several metabolites including Sal B, quercetin, Asp VI, paeoniflorin, verbascoside, catalpol, loganin, hyperoside, and kaempferol were identified (Figure 5) (Wu et al., 2023). Among these, metabolites such as quercetin, paeoniflorin, verbascoside, and catalpol have been reported to attenuate impaired reproductive capacity by suppressing oocyte apoptosis or increasing intestinal function (Martino et al., 2016; Gao et al., 2023; Hua et al., 2023; Sirotkin, 2024; Song, et al., 2024). However, several of these metabolites exhibit toxic effects as their concentrations increase. For instance, in rat intestinal epithelial cells, 80 μM catalpol ameliorates the inflammatory response by activating the AMPK signaling pathway, while 160 μM catalpol significantly reduces cell viability (Wei et al., 2014). Similarly, loganin at 10 μmol/L shows antioxidant function, while concentrations ranging from 25 to 50 μmol/L show no effect on cell viability, with low cytotoxicity observed up to 100 μmol/L (Li et al., 2024). Hyperoside at 50 μg/L significantly induces cell proliferation compared to the control, while 75 μg/L results in reduced proliferation relative to the 50 μg/L treatment, indicating potential cytotoxic effects (Nie et al., 2019). Moreover, 100 μM hyperoside extends lifespan in nematodes, whereas a higher concentration (200 μM) is less effective (Yu et al., 2025). The present study also reveals a bidirectional effect of the BSHX decoction: at 62.5 mg/mL, it significantly improves heat-stress resistance in BPA-exposed nematodes, whereas at 125 mg/mL, it fails to confer such benefits. We hypothesize that at the higher concentration (125 mg/mL), certain metabolites may exert adverse effects on reproduction, the heat stress response, or intestinal function. Nevertheless, the precise dose-response relationships and combinatorial effects of these metabolites warrant further in-depth investigation.

Although LC/Q-TOF detected several metabolites in the decoction samples, only Sal B from *S. miltiorrhiza* Bunge, quercetin from *C. chinensis* Lam., and Asp VI from *D. asper* Wall. ex DC. were identified in BSHX-treated nematodes (Wu et al., 2023). Beyond its antioxidative property, Sal B inhibits osteosarcoma cell growth and triggers apoptosis via the p38 MAPK pathway (Zeng et al., 2018), and has been predicted to target the JNK pathway through network pharmacology and molecular docking (Lei et al., 2021). Additionally, it mitigates mitochondrial dysfunction by enhancing UPR^mt^ and stabilizing the mitochondrial membrane potential (Tao et al., 2017; Chen et al., 2024). Similarly, quercetin enhances the maturation of oocytes *in vitro* by scavenging mitochondrial ROS (Cao et al., 2022), and may inhibit necroptosis by regulating mitophagy and the unfolded protein response (Chang et al., 2024). Asp VI, on the other hand, boosts antioxidant capacity in cardiomyocytes by preventing mitochondrial damage (Li et al., 2010) and alleviates mitochondrial dysfunction in osteoarthritis models (Qiao et al., 2025). Notably, network pharmacology studies suggest that the *Achyranthes bidentata*-*D. asper* herb pair acts on osteoporosis by targeting the core MAPK cascade (ERK/JNK/p38) (Li et al., 2023). Building on this foundation, our study demonstrates that the combination of Sal B, quercetin, and Asp VI enhances fertility through the PMK-1/p38 MAPK pathway and UPR^mt^ (Figures 6, 7). Collectively, these findings indicate that the therapeutic effects of this metabolite combination on fertility likely arise from the regulation of MAPK signaling and mitochondrial pathways.

Next, the optimal combination, i.e., Sal B 50 μg/mL + Que 2.5 μg/mL + Asp VI 10 μg/mL, exhibited the most significant improvement in fertility (Figure 6A). This combination also significant increased heat stress resistance through a pharmacological mechanism resembling that of the BSHX decoction. These results indicate that the three metabolites are key active metabolites of the BSHX decoction, and that their combination contributes substantially to the decoction’s fertility-enhancing effects. Although BSHX contains numerous other metabolites that have not yet been identified or characterized, our study provides a foundation for developing combination therapies to improve fertility.

The experiments in this study were conducted using the model organism *C*. *elegans*. However, *C. elegans* lacks organs such as the liver and kidneys. Given that the liver is the primary organ responsible for drug metabolism, we employed network-toxicology to analyze the potential hepatotoxicity of seven active metabolites in Bushen Huoxue decoction. The results indicated that the seven main metabolites in the decoction may exert hepatotoxicity through 26 genes, and KEGG pathway analysis suggested that the PI3K−Akt signaling pathway is the most likely pathway involved. Among these 26 genes, *XDH* mRNA expression is decreased in human hepatocellular carcinoma (HCC), and its expression can activate the PI3K-Akt signaling pathway, inducing cytotoxic immune responses (Lin et al., 2021). In HCC, the EGFR/PI3K/Akt/mTOR pathway is abnormally activated in approximately 50% of cases, and this dysregulated activation is involved in various cellular processes, including cell proliferation, tumor cell differentiation, autophagy, metabolism, and the epithelial–mesenchymal transition (Sun et al., 2021). Another network pharmacology study has similarly identified PARP1 and XDH as targets of hepatoxicity, with the PI3K-Akt pathway being the primary signaling pathway involved (Wang et al., 2023).

This study primarily utilized *C. elegans* to investigate the pharmacological mechanisms by which BSHX improves fertility. The presence of homologous genes and highly conserved signaling pathways supports the plausibility of the proposed mechanisms. However, given the structural simplicity of the nematode model compared to the complex tissue systems of mammals, validation in mammalian models would strengthen the extrapolation of the findings. This work also explored the combination of major active metabolites derived from the BSHX decoction and demonstrated that the pharmacological mechanism of the metabolite combination closely mirrors that of the full decoction. Nevertheless, Chinese herbal formulas are characterized by their complex metabolites. Future studies could employ network pharmacology to systematically analyze the key metabolites of the formula, followed by experimental validation.

## 5 Conclusion

**FIGURE 10:**
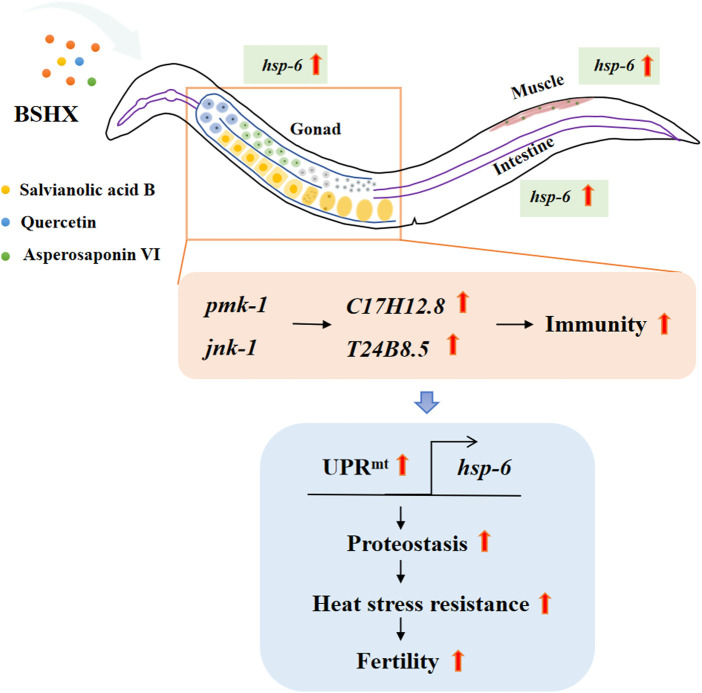
Graphic illustrating the BSHX decoction enhances fertility by modulating systemic stress responses in *C. elegans.* BSHX activates conserved PMK-1 and JNK-1 MAPK signaling pathways in germ cells. Notably, PMK-1 activation upregulates the immune peptide *T24B8.5* and initiates the UPR^mt^, thereby improving proteostasis and oocyte quality.

BSHX decoction significantly enhanced the fertility of BPA-exposed nematodes by suppressing abnormal protein accumulation in proximal oocytes. It conferred heat stress resistance in germ cells through the PMK-1 and JNK-1 MAPK signaling pathways. BSHX upregulated the expression of the PKM-1 target T24B8.5, thereby contributing to improved fertility. The BSHX-induced activation of the PMK-1 MAPK pathway further triggered UPR^mt^ in the gonad and intestine. The UPR^mt^ -regulated gene *hsp-6* played an essential role in maintaining mitochondrial function by reducing mitochondrial ROS levels and increasing mitochondrial membrane potential, ultimately promoting enhanced fertility. Moreover, a combination of metabolites from the BSHX decoction significantly restored fertility in BPA-exposed nematodes through a mechanism highly similar to the whole decoction (the graphical mechanism illustrated in Figure 10).

## Data Availability

The original contributions presented in the study are included in the article/Supplementary Material, further inquiries can be directed to the corresponding author.
