# Supplementary material for: Bushen Huoxue decoction alleviates bisphenol a-induced infertility through the PMK-1 mitogen-activated protein kinases signaling pathway and downstream mitochondrial unfolded protein response in Caenorhabditis elegans
Source: Front Pharmacol. 2026 Jan 15;16:1713681. doi: 10.3389/fphar.2025.1713681 (PMC12852476; doi:10.3389/fphar.2025.1713681)
Supplement: Supplementary file 1 [file Supplementaryfile1.doc]

| **RNAi primer** | | |
| --- | --- | --- |
| Gene name | Forward | Reverse |
| *hsp-6* | cgcggtggcggccgctctagaAATGAGCAGAATGCCAAAGGTG | atcgaattcctgcagcccgggTTAGTTTTGCTCCTTCTTTGGCTC |
| *T24B8.5* | cgcggtggcggccgctctagaAGATTAATTGATAAGAACTCCTCATATAAACTT | atcgaattcctgcagcccgggATTCTTAGTTTCTTGTCTGGTCTGAAAT |
| *pmk-1* | cgcggtggcggccgctctagaTCATTTCAACTGATTTTTACTTTATTTTTC | atcgaattcctgcagcccgggACATGATACGAGTCAATTGATCGATG |
| **qPCR primer** | | |
| Gene name | Forward | Reverse |
| *act-3* | ATCCGTAAGGACTTGTACGCCAAC | GGGCGATGATCTTGATCTTCATGG |
| *mpk-1* | GTATCGACATCGAGCAAGCA | CAGGATTCTGCCCTCCATTA |
| *pmk-1* | AAATGACTCGCCGTGATTTC | CATCGTGATAAGCAGCCAGA |
| *jnk-1* | ACGACGCTGTCTCTTTGGAT | CGTATCAAACGCTGAGCAAA |
| *C17H12.8* | AATACCAGCGGGTGCTAATG | AAATTTTGCAGATCGGCTTG |
| *K08D8.5* | TGTCTCAACTGGCAACAAGC | GCAACAGCAGTCGCATAAAA |
| *T24B8.5* | TCGACTCAAGACCATCATGC | CCACAGATTTGGCAGGTTTT |
| *hsp-6* | CAAGATTGTCAAAGCCAGCA | GCTCGTTGATGACACGAAGA |
| *hsp-60* | TCAGTGACGCCATGAAGAAG | CCGGCACAATATCCTGAACT |
| *ymel-1* | TCGATTACCGAAAGGTGTCC | CACGAGCCTTAGCCTTATCG |
| *clpp-1* | GTCATTCGTGCCGAAGAAAT | TTGATCCGTTGTGAGTCTCG |
| *lonp-1* | TTCCACTCATGACGATCCAA | AGTGAAAGCTGTCGCCTGAT |
